# Risk factors associated with outbreaks of seasonal infectious disease in school settings, England, UK

**DOI:** 10.1017/S0950268820002824

**Published:** 2020-11-18

**Authors:** A. L. Donaldson, J. P. Harris, R. Vivancos, S. J. O'Brien

**Affiliations:** 1 NIHR Health Protection Research Unit in Gastrointestinal Infections, University of Liverpool, Liverpool, UK; 2 Institute of Population Health Sciences, University of Liverpool, Liverpool, UK; 3 Field Epidemiology Service, Public Health England, Liverpool, UK

## Abstract

Children are important transmitters of infection. Within schools they encounter large numbers of contacts and infections can spread easily causing outbreaks. However, not all schools are affected equally. We conducted a retrospective analysis of school outbreaks to identify factors associated with the risk of gastroenteritis, influenza, rash or other outbreaks. Data on reported school outbreaks in England were obtained from Public Health England and linked with data from the Department for Education and the Office for Standards in Education, Children's Services and Skills (Ofsted). Primary and all-through schools were found to be at increased risk of outbreaks, compared with secondary schools (odds ratio (OR) 5.82, 95% confidence interval (CI) 4.50–7.58 and OR 4.66, 95% CI 3.27–6.61, respectively). School size was also significantly associated with the risk of outbreaks, with higher odds associated with larger schools. Attack rates were higher in gastroenteritis and influenza outbreaks, with lower attack rates associated with rashes (relative risk 0.17, 95% CI 0.15–0.20). Deprivation and Ofsted rating were not associated with either outbreak occurrence or the subsequent attack rate. This study identifies primary and all-through schools as key settings for health protection interventions. Public health teams need to work closely with these schools to encourage early identification and reporting of outbreaks.

## Introduction

Children are recognised as important transmitters of seasonal and pandemic infectious disease [1–3]. They have more naïve immune systems which increase susceptibility to infection and are commonly acknowledged to have poorer levels of hand and respiratory hygiene. Within schools, they experience a large number of contacts with peers [1], and infections can spread easily through direct and indirect transmission [4, 5]. Unlike healthcare settings, schools do not have standard infection control practices and may have inadequate cleaning programmes or lack the facilities to support proper handwashing [6]. Furthermore, infection control is not a key focus for schools or teachers and implementing hygiene interventions can be challenging [7]. These factors result in an increased risk of outbreaks within school settings.

Rashes, gastrointestinal and respiratory infections are common causes of outbreaks within schools [8–11]. Outbreaks need to be managed promptly to prevent further spread of infection both within the school environment and outward to households and the wider community [12]. Measures such as exclusion, environmental cleaning, hand washing and promoting good respiratory hygiene can be used to control outbreaks [5, 13]. Public health agencies can offer advice and support to schools on the management of outbreaks, but it is likely that many outbreaks go unrecognised and unreported [14, 15].

Not all schools are affected equally by outbreaks, and there is evidence that outbreaks and subsequent attack rates are influenced by the age of pupils and school size [5, 9, 16]. Socioeconomic status may influence disease risk and severity [17, 18] as well as affecting vaccine uptake [19] which could contribute towards the risk of outbreaks in school settings. This study seeks to identify factors associated with outbreak occurrence and attack rate in schools. Understanding which factors increase the risk of outbreaks could help identify higher-risk schools and support targeted interventions and training to help prevent future outbreaks.

## Methods

### Study population

The study population was schools in England, UK. There are just under 24 000 registered schools in England, which include state-schools, academies, independent/private schools, special schools for children with special educational needs and pupil referral units (PRUs) for children who are not able to attend a mainstream school [20]. Together, these schools cover a pupil population of 8.8 million. Schools teach primary education (ages 4–11 years), secondary education (ages 11–18 years) or both (all-through schools).

### Data sources

Data on reported school outbreaks in England are held by Public Health England (PHE). Outbreaks in school settings are self-reported to PHE by schools, in line with national guidance for health protection in schools [21]. An outbreak is defined as two or more cases linked in time or place, or a greater than expected rate of infection compared with the usual background rate for a given place and time [21]. Schools are advised to contact PHE as soon as an outbreak is suspected, although reporting is not mandatory. The decision to declare an outbreak is made by PHE and the school is advised on appropriate infection control measures to manage the outbreak. Such measures include hand hygiene, respiratory hygiene, environmental cleaning, exclusion and letters to parents [21]. Data on reported school outbreaks across England were extracted from the PHE database for the 2016/2017 and 2017/2018 academic years. The academic year was defined as running from 1 September to the 31 August. Outbreaks linked to nurseries, universities or colleges for those over 18 years of age, care homes for children, households, community settings or visitor attractions were excluded from the dataset. Special schools and PRUs were included.

Outbreak data were combined with nationally available data from the Department for Education and the Office for Standards in Education, Children's Services and Skills (Ofsted) [22, 23]. The Department for Education provides routine data on the demographics and performance of registered schools, and Ofsted publishes data on school inspections. Ofsted routinely inspect all state-registered schools and assesses them according to pupil outcomes, quality of teaching, learning and assessment, effectiveness of leadership and management, and personal development, behaviour and welfare. Schools are then given an overall effectiveness score which ranges from 1–4 (outstanding to inadequate). Ofsted also provides a deprivation quintile for each school, which is calculated from the Index of Multiple Deprivation (IMD) for the postcode of residence of each pupil [24]. The IMD scores are then averaged for the school to give an overall score and schools are placed into quintiles; quintile one representing the least deprived and quintile five representing the most deprived.

### Statistical methods

The unique reference number was identified for each school within the outbreak dataset and used to link PHE data with data from the Department for Education and Ofsted, for the corresponding academic year. Descriptive statistics were used to explore seasonal trends in outbreaks and variations in the number and proportion of outbreaks, broken down by different explanatory variables. Variables were selected based on the experience of the research group and the availability of national school-level data. The explanatory variables included the size of the school, phase of education, Ofsted score, deprivation index and the gender of the school (single sex *vs.* mixed). The size of school was included as a categorical variable, the categories determined by the distribution of the data. Phase of education was categorised as primary, secondary and all-through schools (covering both primary and secondary year groups). Multivariable logistic regression was used to identify factors associated with schools which had experienced an outbreak, compared with schools which had not experienced an outbreak. Associations were compared for schools with one outbreak and schools with two or more outbreaks over the study period. Modelling was repeated, stratified by outbreak cause, to explore differences in the predictors of outbreak occurrence for the major causes of outbreaks. Cramer's *V* coefficients were used to identify any significant correlations between the explanatory variables which could affect the regression model and variance inflation factors (VIF) were used to check the model for multicollinearity.

Attack rates were calculated by dividing the number of symptomatic pupils by the total number in the school, presented as a rate per 100 pupils/year. Descriptive statistics were used to explore variations in attack rate across the different explanatory variables. Explanatory variables included the size of the school, phase of education, Ofsted score, deprivation index, as well as the cause of the outbreak and the delay in reporting. Size of school and phase of education were categorised as described above. The cause of the outbreak was broken down into gastroenteritis, influenza, rash or other. Delay in reporting was calculated as the difference in days between the date of onset of the index case and the date the outbreak was reported to PHE. As delay in reporting was skewed, a sensitivity analysis was undertaken, removing major and minor outliers to explore the impact of skewed data on model performance. Major outliers were defined as datapoints falling more than three times the interquartile range above the third quartile, and minor outliers falling one and a half times the interquartile range above the third quartile. Quasi-Poisson regression was used to identify factors associated with attack rate for all outbreaks and outbreaks stratified by cause. This was chosen instead of a Poisson regression due to the high level of variation within the count data. Quasi-Poisson methods relax the assumption that the variance is equal to the mean and allows for more robust calculation of confidence intervals (CIs).

Odds ratios (ORs) and 95% CIs were calculated from the logistic regression models. Relative risk (RR) with corresponding CIs was generated from the quasi-Poisson regression modelling. All statistical analyses were undertaken in R 3.3.2 [25].

## Results

**Fig. 1.**
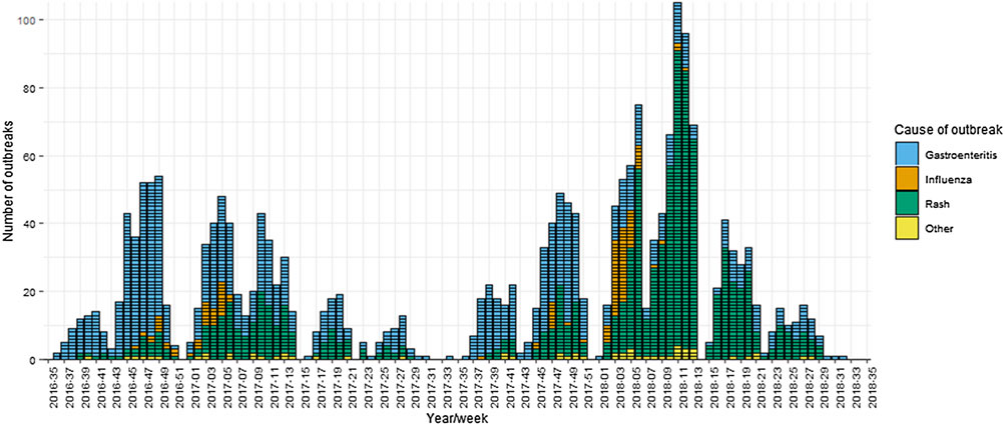
School outbreaks reported to Public Health England by week and cause, 1 September 2016–31 August 2018.

From 1 September 2016 to 31 August 2018, there were 2207 outbreaks in schools reported to PHE. Of these, 90 were excluded as they did not meet the inclusion criteria. Gastroenteritis was the most common cause of reported outbreaks, accounting for 47% (*n* = 998). This was followed by rash (44%, *n* = 935) and influenza (6%, *n* = 126). Other causes of outbreaks accounted for less than 1% each and included respiratory tract infections, conjunctivitis, hepatitis, impetigo, infestations and worms. Outbreaks ranged in size from 2 to 300 cases, with a median of 10 cases per outbreak. There were clear seasonal trends in reported outbreaks, as shown in Figure 1. Across both academic years, gastroenteritis outbreaks peaked between November and January, followed by a peak in rashes between January and March. In 2017/2018, the peak in rash outbreaks was particularly dominant, driving a higher number of reported outbreaks that year.

Outbreaks occurred in primary, secondary and all-through schools. Schools ranged in size from 11 pupils to 2170, with a median size of 299 pupils. Only 1% of outbreak schools were single sex schools, so this was dropped as a covariate in the regression analysis. One outbreak occurred in a PRU, which was subsequently excluded due to a lack of available national data on PRUs. Ofsted ratings were available for 1731 (94%) of the schools. The scores in the sub-categories tended to be consistent with the overall score, therefore only the overall Ofsted rating was included in the analysis.

### Outbreak occurrence

A total of 1841 schools experienced at least one outbreak across the study period and 232 (12.6%) reported more than one outbreak. Primary schools accounted for 86% of outbreak schools, whilst 7% were all-through schools and 6% were secondary schools (Table 1). There was evidence of correlation between school size and phase of education (Cramer *V* = 0.52), but no other correlations were found between the explanatory variables. There was no evidence of multicollinearity in the regression models (VIF < 2).

**Table 1.** Summary of the number and proportion of outbreaks, by cause and explanatory variable

Explanatory variables	All schools *n* (%)	All outbreaks *n* (%)	Gastroenteritis *n* (%)	Rash *n* (%)	Influenza *n* (%)	Other *n* (%)
Total number of outbreaks	/	2116	997	935	126	58
Total number of schools	26 985	1841	923	859	124	57
School size						
≤50	1339 (5.0%)	20 (1.1%)	15 (1.6%)	3 (0.3%)	2 (1.6%)	0 (0%)
51–200	6878 (25.5%)	413 (22.4%)	236 (25.6%)	162 (18.9%)	17 (13.7%)	10 (17.5%)
201–400	8578 (31.8%)	727 (39.5%)	359 (38.9%)	360 (41.9%)	40 (32.3%)	20 (35.1%)
401–600	4047 (15.0%)	396 (21.5%)	185 (20.0%)	212 (24.7%)	30 (24.2%)	8 (14.0%)
>600	3879 (14.4%)	248 (13.5%)	113 (12.2%)	106 (12.3%)	32 (25.8%)	15 (26.3%)
Not known	2264 (8.4%)	37 (2.0%)	15 (1.6%)	16 (1.9%)	3 (2.4%)	4 (7.0%)
Phase of education						
Primary	20 151 (74.8%)	1591 (86.4%)	784 (84.9%)	804 (93.6%)	72 (58.0%)	37 (64.9%)
Secondary	4498 (16.7%)	114 (6.2%)	61 (6.6%)	18 (2.1%)	28 (22.6%)	14 (24.6%)
All-through	2275 (8.4%)	136 (7.4%)	78 (8.5%)	37 (4.3%)	24 (19.4)	6 (10.5%)
Not known	61 (0.2%)	0 (0%)	0 (0%)	0 (0%)	0 (0%)	0 (0%)
Ofsted overall rating						
1 – Outstanding	4598 (17.0%)	372 (20.2%)	179 (19.4%)	175 (20.4%)	25 (20.2%)	13 (22.8%)
2 – Good	15 867 (58.8%)	1130 (61.4%)	564 (61.1%)	550 (64.0%)	59 (47.6%)	30 (52.6%)
3 – Requires improvement	2567 (9.5%)	180 (9.8%)	100 (10.8%)	77 (9.0%)	15 (12.1%)	3 (5.3%)
4 – Inadequate	1085 (4.0%)	49 (2.7%)	27 (2.9%)	17 (2.0%)	5 (4.0%)	2 (3.5%)
NA	2868 (10.6%)	110 (6.0%)	53 (5.7%)	40 (4.7%)	20 (16.1%)	9 (15.8%)
Deprivation						
1 – Least deprived	4370 (16.2%)	316 (17.2%)	149 (16.1%)	147 (17.1%)	29 (23.4%)	13 (22.8%)
2	4483 (16.6%)	338 (18.4%)	158 (17.1%)	176 (20.5%)	18 (14.5%)	7 (12.3%)
3	4543 (16.8%)	365 (19.8%)	198 (21.5%)	168 (19.6%)	16 (12.9%)	7 (12.3%)
4	4670 (17.3%)	334 (18.1%)	173 (18.7%)	157 (18.3)	19 (15.3%)	10 (17.5%)
5 – Most deprived	4539 (16.8%)	357 (19.4%)	182 (19.7%)	164 (19.1%)	16 (12.9%)	10 (17.5%)
Not known	4380 (16.2%)	131 (7.1%)	63 (6.8%)	47 (5.5%)	26 (21.0%)	10 (17.5%)

Primary and all-through schools were found to be at increased risk of an outbreak, compared with secondary schools (OR 5.82, 95% CI 4.50–7.58 and OR 4.66, 95% CI 3.27–6.61, respectively). Outbreak occurrence was also significantly associated with school size, with the odds of an outbreak increasing as school size increased (Table 2). The occurrence of an outbreak was not associated with Ofsted rating or the level of deprivation in a school. Similar associations were found for the occurrence of multiple outbreaks and across both gastroenteritis and rash outbreaks. The number of influenza outbreaks were insufficient to allow separate analysis.

**Table 2.** Factors associated with the odds of an outbreak in a school, by cause; a multivariable logistic regression model

Explanatory variables	All outbreaks OR (95% CI)	Gastroenteritis OR (95% CI)	Rash OR (95% CI)
Phase of education			
Secondary	1	1	1
Primary	5.82 (4.50–7.58)	4.53 (3.19–6.54)	18.31 (11.17–31.94)
All-through	4.66 (3.27–6.61)	4.76 (3.01–7.49)	6.56 (3.13–13.54)
School size			
≤50	0.26 (0.14–0.42)	0.43 (0.22–0.76)	0.08 (0.01–0.24)
51–200	0.77 (0.68–0.88)	0.94 (0.79–1.12)	0.62 (0.51–0.75)
201–400	1	1	1
401–600	1.23 (1.07–1.40)	1.07 (0.88–1.29)	1.44 (1.20 –1.72)
>600	1.80 (1.48–2.18)	1.46 (1.10–1.91)	2.26 (1.73–2.91)
Ofsted rating			
1 – Outstanding	1	1	1
2 – Good	0.89 (0.79–1.02)	0.91 (0.76–1.08)	0.93 (0.78–1.12)
3 – Requires improvement	1.06 (0.87–1.29)	1.13 (0.87–1.47)	1.09 (0.82–1.43)
4 – Inadequate	0.92 (0.64–1.29)	1.19 (0.75–1.80)	0.64 (0.32–1.13)
Deprivation index			
1 – Least deprived	1	1	1
2	1.05 (0.89–1.23)	1.01 (0.80–1.27)	1.18 (0.94–1.49)
3	1.08 (0.92–1.27)	1.27 (1.02–1.58)	1.01 (0.80–1.28)
4	0.89 (0.75–1.05)	1.05 (0.83–1.32)	0.82 (0.64–1.05)
5 – Most deprived	0.95 (0.81–1.13)	1.09 (0.87–1.38)	0.86 (0.67–1.09)

OR, odds ratio; CI, confidence interval.

### Attack rates

Of 2116 outbreaks, 1813 (86%) had data on attack rates. Attack rates ranged from 0.1 per 100 pupils to 74.2 per 100, with a median attack rate of 3.6 per 100 pupils. Attack rates varied depending on the cause of the outbreak, with the highest median attack rate occurring for gastroenteritis outbreaks (8.3 per 100) and the lowest for rash (1.1 per 100) (Table 3). Median attack rate decreased as school size increased but the number of cases did not vary accordingly, with all but the smallest school size having a median of 9–10 cases per outbreak. Delays in reporting varied from zero (outbreak reported same day as onset) to 105 days. The median delay in reporting was 3 days. This was similar for gastroenteritis outbreaks (3 days), rash (4 days) and influenza (4 days). In addition to the correlation identified between phase of education and school size (Cramer's *V* = 0.42), there was some evidence of association between the cause of outbreak and delay in reporting (Cramer's *V* = 0.34). No other correlations were identified between the explanatory variables. There was no evidence of multicollinearity in the regression model (VIF < 2.5).

**Table 3.** Summary of the number of cases, number at risk and attack rate, by explanatory variable

Explanatory variable	Number of symptomatic cases median (IQR)	Number at risk median (IQR)	Attack rate median (IQR)
School size			
≤50	4 (3–7)	36 (27–48)	11.8 (8.6–22.5)
51–200	9 (3–20)	134 (96–175)	7.2 (3.0–16.7)
201–400	10 (3–23)	262 (218–335)	3.5 (1.2–9.3)
401–600	10 (3–24)	442 (419–481)	2.4 (0.7–5.8)
>600	10 (3–30)	717 (630–949)	1.4 (0.4–5.8)
Phase of education			
Primary	10 (3–22)	280 (197–420)	3.6 (1.2–9.6)
Secondary	12 (4–40)	917 (580–1200)	2.6 (0.4–10.3)
All-through	8 (3–20)	285 (122–471)	4.2 (1.4–10.1)
Cause			
Gastroenteritis	20 (11–33)	248 (173–419)	8.3 (4.2–15.2)
Influenza	23 (13–44)	392 (230–579)	7.1 (3.7–11.7)
Rash	3 (2–5)	330 (209–450)	1.1 (0.6–2.3)
Other	7 (3–12)	232 (144–610)	2.5 (0.9–6.7)
Ofsted overall rating			
1 – Outstanding	9 (3–22)	289 (179–472)	3.4 (1.1–9.7)
2 – Good	10 (3–22)	277 (190–420)	3.7 (1.2–10.0)
3 – Requires improvement	10 (3–25)	331 (216–447)	3.7 (1.2–8.6)
4 – Inadequate	10 (4–24)	310 (221–650)	2.8 (1.1–8.2)
Deprivation			
1 – Least deprived	10 (3–20)	213 (120–401)	4.0 (1.6–11.2)
2	7 (3–22)	251 (175–408)	3.7 (1.2–10.5)
3	10 (3–23)	270 (186–421)	3.5 (1.2–9.8)
4	10 (4–25)	349 (213–455)	3.4 (1.1–9.0)
5 – Most deprived	11 (4–24)	360 (230–471)	3.5 (1.0–8.5)

Attack rate per 100 pupils/year; IQR, interquartile range.

Attack rates were significantly lower in rash and ‘other’ outbreaks compared with gastroenteritis (RR 0.17, 95% CI 0.15–0.20 and RR 0.62, 95% CI 0.37–0.99, respectively), but there was no difference between influenza and gastroenteritis outbreaks (Table 4). School size was also associated with attack rate, with attack rates decreasing as school size increased. Primary schools had higher attack rates compared with secondary schools for gastroenteritis and rash outbreaks, but not for all-cause outbreaks. Attack rate was found to increase marginally with each additional day delay in reporting (RR 1.01, 95% CI 1.00–1.02). This association remained after removing major and minor outliers from the delay in reporting variable but was not significant for gastroenteritis outbreaks. Neither deprivation nor Ofsted score were associated with attack rate. The number of influenza outbreaks were too few to allow separate analysis.

**Table 4.** Factors associated with outbreak attack rate in schools, by cause; a multivariable quasi-Poisson regression model

Explanatory variables	All outbreaks RR (95% CI)	Gastroenteritis RR (95% CI)	Rash RR (95% CI)
Phase of education			
Secondary	1	1	1
Primary	1.15 (0.93–1.41)	1.30 (1.00–1.71)	2.56 (1.08–7.44)
All-through	0.92 (0.66–1.27)	0.87 (0.56–1.31)	1.44 (0.32–5.74)
School size			
≤50	2.82 (1.22–5.45)	3.15 (1.18–6.69)	5.62 (0.32–25.33)
51–200	1.58 (1.37–1.82)	1.53 (1.29–1.82)	1.82 (1.37–2.40)
201–400	1	1	1
401–600	0.74 (0.65–0.85)	0.74 (0.62–0.87)	0.70 (0.55–0.90)
>600	0.69 (0.58–0.83)	0.76 (0.61–0.95)	0.38 (0.25–0.56)
Cause			
Gastroenteritis	1	–	–
Influenza	1.00 (0.85–1.17)	–	–
Rash	0.17 (0.15–0.20)	–	–
Other	0.62 (0.37–0.99)	–	–
Ofsted rating			
1 – Outstanding	1	1	1
2 – Good	0.97 (0.85–1.11)	0.89 (0.76–1.05)	0.91 (0.71–1.20)
3 – Requires improvement	0.99 (0.82–1.19)	0.90 (0.70–1.14)	0.90 (0.60–1.34)
4 – Inadequate	0.73 (0.49–1.05)	0.80 (0.49–1.23)	0.94 (0.41–1.88)
Deprivation index			
1 – Least deprived	1	1	1
2	1.00 (0.86–1.19)	1.05 (0.85–1.30)	1.16 (0.84–1.61)
3	0.98 (0.83–1.16)	0.98 (0.80–1.21)	1.15 (0.83–1.60)
4	1.09 (0.92–1.29)	1.12 (0.91–1.39)	1.31 (0.93–1.85)
5 – Most deprived	1.12 (0.95–1.33)	1.09 (0.88–1.35)	1.51 (1.04–2.18)
Delay in reporting			
Per additional day	1.01 (1.00–1.02)	1.00 (0.99–1.01)	1.03 (1.02–1.03)

RR, relative risk; CI, confidence interval.

## Discussion

In this study, across a 2-year period, outbreaks occurred in almost 1 in 10 schools. The most common causes of reported outbreaks were gastroenteritis and rashes, with the majority of outbreaks occurring in primary schools. Primary and all-through schools, as well as larger school size were associated with an increased risk of outbreaks, whilst Ofsted rating and deprivation were not associated with outbreak occurrence. Attack rates were higher in smaller schools and with each additional day delay in reporting. Lower attack rates were associated with larger schools and outbreaks caused by rashes.

The finding that primary schools were disproportionately affected by outbreaks is consistent with existing literature [5, 9, 26, 27]. Younger children are known to have a greater vulnerability to infection, higher virus shedding and poorer levels of hand and respiratory hygiene which increase the risk of illness [1]. The effect of school size on outbreak occurrence mirrors the risk of infection by household size and care home size [28–30]. The increased outbreak risk in larger schools could be attributed to more pupils entering the school environment and therefore more opportunity for infection to be introduced into the school. Larger schools also experienced lower attack rates, a phenomenon observed in other settings [16, 30]. In this study, this finding is most likely driven by changes in the attack rate denominator. The median number of cases did not vary significantly between schools of different sizes, but the large differences in the number of children at risk result in larger schools having significantly lower attack rates for the same number of cases. It may be that class size, rather than school size, is a more important variable for disease transmission in schools and studies have shown the majority of children's close contacts in school are within their immediate class [31, 32]. These contacts have a high potential for disease transmission, as children are in close proximity to their classmates for prolonged periods of time. Unfortunately, data on class sizes were not available to include in this study, but in England class size is limited to approximately 30 children [33], so for the majority of schools the class size will be similar. Even small schools may combine year groups to increase otherwise small class sizes. This offers a potential explanation for why similar numbers of cases were observed regardless of the overall size of the school.

Early detection and reporting of outbreaks is crucial for the implementation of control measures and the finding that attack rate increases with delays in reporting is consistent with findings from care home settings [30]. However, the association was borderline and for schools this relationship is likely to be affected by school holidays, which create a natural break in transmission and help control and terminate outbreaks without additional intervention. The effect of school holidays on the size and timing of seasonal outbreaks is a topic for further investigation.

Of note in this study was the relatively small proportion of influenza outbreaks reported over a 2-year period (*n* = 126). This is in strong contrast to the numbers reported in other years within the UK [9]. Influenza strains vary year on year [34], and so the years included in this study may have captured milder influenza seasons and consequently underestimate the role of influenza in school outbreaks. However, in 2013 the UK started the phased introduction of the universal childhood influenza vaccination [35], and as the number of children receiving the vaccine increase, influenza may become a less frequent cause of school outbreaks. Vaccine uptake, and factors which influence vaccine uptake, may then have an increasing role in the occurrence of school outbreaks. This could alter the importance of deprivation, which was not found to be associated with either outbreak occurrence or attack rate in this study, but has been linked to the uptake of influenza vaccination [19, 36].

### Limitations

Schools do not have a statutory duty to report outbreaks to PHE and therefore, this study may underestimate the total number of outbreaks occurring. As outbreaks are self-reported, these data may be subject to reporting bias which could impact on the strength of associations within the analysis. Unreported outbreaks are most likely to have small number of cases, which do not cause significant disruption to the school. This could impact on the association between delay in reporting and attack rate, as such outbreaks are likely to have low attack rates and yet unmeasurable delays in reporting. Furthermore, the distribution of delays in reporting was skewed, which could affect the regression model performance. However, removing major and minor outliers from this variable did not alter the association between delay in reporting and attack rate, suggesting the skewed data had minimal impact on the results.

A further limitation of the data is the variation in how outbreaks in schools are recorded and followed up. The initial documentation of an outbreak includes the number of symptomatic cases at the time the outbreak is declared, and this will be updated each time contact is made with the school. However, smaller outbreaks which resolve quickly may not necessitate further contact with the school beyond the initial report. Therefore, it is possible that additional cases occurring towards the end of outbreaks may have been missed. Whilst the number of additional cases is likely to be small, the case numbers within the PHE dataset may underestimate the total number of children affected and consequently the attack rates represent a conservative estimate.

Finally, each year different organisms may have a greater or lesser role in driving outbreaks in schools. Organism strains vary and some years will have a greater impact on children and schools than others. Consequently, this analysis is not a definitive assessment of the impact of infectious diseases on schools and ongoing timely data on outbreaks is required to detect the key organisms affecting children.

## Conclusion

This study has identified primary and all-through schools as being at increased risk of outbreaks and therefore health protection interventions need to focus on these settings. Larger schools were also at increased risk and need to ensure they are aware of the importance of infection prevention measures such as handwashing and environmental cleaning. Gastroenteritis and influenza outbreaks were associated with higher attack rates and public health teams need to work closely with schools to encourage early identification and reporting of these outbreaks.

## Data Availability

The outbreak data that support the findings of this study are available on request from Public Health England. Department for Education school performance data are openly available at GOV.UK, Find and Compare Schools in England (https://www.gov.uk/school-performance-tables). Ofsted data are openly available at GOV.UK, Research and Statistics (https://www.gov.uk/search/research-and-statistics).
